# Quality and uptake of antenatal and postnatal care in Haiti

**DOI:** 10.1186/s12884-016-1202-7

**Published:** 2017-02-02

**Authors:** Kelsey R. Mirkovic, Eva Lathrop, Erin N. Hulland, Reginald Jean-Louis, Daniel Lauture, Ghislaine Desinor D’Alexis, Endang Hanzel, Reynold Grand-Pierre

**Affiliations:** 1 Epidemic Intelligence Service, Centers for Disease Control and Prevention, Atlanta, GA USA; 2 Division of Global Health Protection, Centers for Disease Control and Prevention, Atlanta, GA USA; 3 Division of Global Health Protection, Centers for Disease Control and Prevention, Port-au-Prince, Haiti; 4 Haiti Ministry of Public Health and Population, Port-au-Prince, Haiti

**Keywords:** Antenatal care, Postnatal care, Counseling, Haiti

## Abstract

**Background:**

Despite improvement, maternal mortality in Haiti remains high at 359/100,000 live births. Improving access to high quality antenatal and postnatal care has been shown to reduce maternal mortality and improve newborn outcomes. Little is known regarding the quality and uptake of antenatal and postnatal care among Haitian women.

**Methods:**

Exit interviews were conducted with all pregnant and postpartum women seeking care from large health facilities (*n* = 10) in the Nord and Nord-Est department and communes of St. Marc, Verrettes, and Petite Rivière in Haiti over the study period (March-April 2015; 3–4 days/facility). Standard questions related to demographics, previous pregnancies, current pregnancy, and services/satisfaction during the visit were asked. Total number of antenatal visits were abstracted from charts of recently delivered women (*n* = 1141). Provider knowledge assessments were completed by antenatal and postnatal care providers (*n* = 39). Frequencies were calculated for descriptive variables and multivariable logistic regression was used to explore predictors of receiving 5 out of 10 counseling messages among pregnant women.

**Results:**

Among 894 pregnant women seeking antenatal care, most reported receiving standard clinical service components during their visit (97% were weighed, 80% had fetal heart tones checked), however fewer reported receiving recommended counseling messages (44% counselled on danger signs, 33% on postpartum family planning). Far fewer women were seeking postnatal care (*n* = 63) and similar service patterns were reported. Forty-three percent of pregnant women report receiving at least 5 out of 10 counseling messages. Pregnant women on a repeat visit and women with greater educational attainment had greater odds of reporting having received 5 out of 10 counseling messages (2^nd^ visit: adjusted odds ratio [aOR] =1.70, 95% confidence interval [CI]: 1.09–2.66; 5+ visit: aOR = 5.44, 95% CI: 2.91–10.16; elementary school certificate: aOR = 2.06, 95% CI: 1.17–3.63; finished secondary school or more aOR = 1.97, 95% CI = 1.05–3.02). Chart reviews indicate 27% of women completed a single antenatal visit and 36% completed the recommended 4 visits.

**Conclusions:**

Antenatal and postnatal care uptake in Haiti is sub-optimal. Despite frequent reports of provision of standard service components, counseling messages are low. Consistent provision of standardized counseling messages with regular provider trainings is recommended to improve quality and uptake of care in Haiti.

## Background

The maternal mortality ratio (MMR) in Haiti is estimated to be 359/100,000 live births [1], and while this represents a 43% decrease from the 1990 estimate of 625/100,000 live births, it remains the highest MMR in the Latin American and Caribbean region and on par with several countries in sub-Saharan Africa. Improvements in access to and quality of intrapartum care, specifically basic and comprehensive emergency obstetrical care, will lead to the largest reductions in maternal death. Both high quality antenatal care and postpartum care with an emphasis on identifying complications and offering family planning methods to achieve healthy birth spacing, can contribute to reductions in maternal deaths [2].

The majority of pregnant women in Haiti receive at least one antenatal visit (90%), however far fewer access the recommended 4 visits (67%) [3]. Anecdotal evidence suggests that care is variable in terms of service components and quality, however there are no systematic data addressing quality of antenatal care in Haiti. Standardized, high quality antenatal care could potentially influence a pregnant woman’s satisfaction with care, her knowledge of danger signs in pregnancy, her decision to seek a facility based delivery and her knowledge of available postpartum family planning methods [4]. Much less is known about the quality and uptake of postnatal care. Only 30% of women and only 19% of infants received any postnatal care in the first 2 days after delivery [3]. Little is known about the services provided at these visits and there are no current estimates of postnatal care uptake at the recommended 1-week or 6-weeks visits.

In order to ensure comprehensive, high quality antenatal and postnatal care, it is imperative to characterize the current quality of service delivery in the realms of antenatal and postpartum care and to better understand the gaps in completeness of care in accordance with the World Health Organization (WHO) recommended guidelines [5]. To address this, a study assessing quality and uptake of antenatal and postnatal care in large health facilities in three departments within Haiti was conducted.

## Methods

### Study sites

All public and private clinics who provide antenatal services to approximately 100 women per week within three target areas of Haiti (Nord department, Nord-Est department, and the communes of St Marc, Verrettes, and Petite Rivière within the Artibonite department– referred to as the Artibonite sub-region for the remainder of this manuscript– were included in the study. These regions were conveniently selected and represent the regions where most CDC provided technical assistance related to maternity care has occurred in Haiti. The approximate number of women seen weekly was estimated using data from the publicly available Monitoring Evaluation and Surveillance Intégrée (MESI). Ten health facilities met the above criteria and were included in the study (2 in Nord department, 3 in Nord-Est department, and 5 in Artibonite sub-region). These ten studies represent an exhaustive sample of antenatal care clinics servicing 100 women per week or more in our three selected regions.

### Data collection

Three data collection methods were used in this study 1 exit interviews with women seeking antenatal or postnatal care, 2) antenatal chart abstraction and 3) provider knowledge assessments. This study uses data from these three methods to triangulate quality of antenatal and postnatal care. Each method is described below.

#### Exit interviews

Exit interviews were conducted in Haitian Creole with pregnant or postpartum women as they exited the facility after their antenatal or postnatal care visit. Four days were spent at each of the Nord and Nord-Est department health facilities (except one health facility in Nord-Est where the clinic is open only 2 days per week) and 3 days were spent at each of the Artibonite health facilities between March-April, 2015. This duration was deemed sufficient to capture a sample of approximately 400 women seeking antenatal care from each department (Nord, Nord-Est, and Artibonite sub-region) for a total of 1200 women. The sample size of 400 women per department was calculated based on an anticipated 10% change in services provided from baseline to endline, assuming a baseline frequency of 50%, a power of 80%, a 95% confidence interval and an anticipated non-response rate of 3%. The sample size of 400 women within each department was distributed population proportional to estimated size among all included health facilities, resulting in a representative sample of women seeking antenatal care from large health facilities by department. All women who were seeking antenatal or postnatal care services on study days were approached for inclusion and nearly the same number of women recorded in the antenatal or prenatal registrar were approached on any given day. Interviews took approximately 10 min to complete and questions included standardized information on demographics, previous pregnancies, and current pregnancy, with a focused section on services and satisfaction regarding the visit they had just completed. Data was entered directly onto Samsung Galaxy 10^®^ tablets (Seoul, South Korea) using Open Data Kit (ODK; open-source). Data were transferred off tablets onto a password protected centralized computer daily. Data were converted to a comma separated value (csv) file using ODK briefcase 10. No personally identifiable information was collected and only women who gave verbal consent were interviewed.

#### Chart abstraction

A sample of individual patient charts was obtained by acquiring the antenatal register used 9 months prior to the data collection period and compiling a list of women seen for antenatal care services starting on the first day of the month and continuing sequentially for 1–3 days of chart abstraction per facility. A minimum of 100 antenatal charts were attempted from all health facilities, however the difficulty and time required to abstract information from a patient chart varied by health facility as a result of differences in chart recording practices and chart storage practice. This number was selected to ensure at least 400 charts from each region were abstracted to approximately match the antenatal care exit interview sample size. From the most recent pregnancy identified in the patient chart, the total number of antenatal care visits was recorded on a paper form which was then entered daily onto a Samsung Galaxy 10 tablet using ODK. Data were transferred off tablets onto a password protected centralized computer daily. Data were converted to a csv file using ODK briefcase 10. Lab-only visits and result-return visits were not included in the total count.

#### Provider knowledge assessment

Knowledge questionnaires were administered to all providers who delivered antenatal and/or postnatal care to women during any of the site visit days. The number of providers varied by facility (*n* = 2–6). Verbal consent was obtained and a questionnaire written in Haitian Creole was completed by the provider. No personally identifiable information was collected. Provider assessments were scored by a single study staff member and data was entered directly into an excel database.

### Data analysis

All analyses were performed using survey procedures in SAS Version 9.3 for Windows (SAS Institute Inc.; Cary, North Carolina; USA). Answers of “I don’t know” were recoded to “no”, except where specified. Weighted data are presented as unweighted frequencies and weighted percentages, except where specified.

### Antenatal care (ANC)

Weights for overall antenatal exit interviews were calculated based on the number of days of data collection at a facility and the response rate within each department. For family planning questions, spontaneous answers were recorded. Patients missing observations for any outcome were excluded from all analyses; to determine how those patients who were excluded differed from the included sample, chi-square tests and Fisher’s exact test for categorical outcomes and *t*-test for continuous outcomes were conducted to assess for significant differences between the two groups.

A total of 10 counseling messages were expected during each visit. A point system was applied to counseling messages such that receipt of each message provided a single point. Univariable and multivariable logistic regression were used to examine determinants of reporting having received at least five of ten standard counseling services during the antenatal care visit. Multivariable models were adjusted for demographics with a known impact on quality of antenatal care services including age, marital/co-habiting status, and education. Prenatal visit number and gestational age were included to assess how repeat visit or gestational age may impact the delivery of recommended counseling messages. Parity was included to assess whether women attending care for a later pregnancy may receive different messaging, and department where services were received (Nord, Nord-Est, Artibonite) was included to account for potential differences in antenatal care across the country.

#### Postnatal care (PNC)

Weights for postnatal exit interviews were calculated based on the number of days of data collection at a facility and the response rate within each department. Patients missing observations for any outcome were excluded from all analyses; to determine how those patients who were excluded differed from the included sample, chi-square tests and Fisher’s exact test for categorical outcomes and *t*-test for continuous outcomes were conducted to assess for significant differences between the two groups.

#### Antenatal care chart abstraction

A total of 1,411 charts were abstracted from the ten study facilities. Weights for the charts were calculated based on the number of women seeking antenatal care in each facility.

#### Provider knowledge questionnaire

An exhaustive survey of providers was conducted in each of the facilities, however only those providers who reported delivering antenatal care were analyzed for the questions on antenatal care knowledge; similarly, only those providers who reported delivering postnatal care were analyzed for the questions on postnatal care knowledge. One provider who did not specify which services he or she provided was excluded from all analyses. All questions were scored with correct answers receiving one point and missing answers were marked as incorrect. All presented data are unweighted due to the small sample size, and thus the inability to make inference on these findings.

## Results

### Antenatal care

Out of 1,049 pregnant women who were approached for an exit interview, 155 refused, yielding a response rate of 85.23%. Out of the 894 women interviewed, 24 were excluded for missing gestational age and 3 were excluded for missing education information resulting in a final analytic sample of 867 pregnant women. Women who were excluded were more likely to be married. Among the remaining 867 pregnant women, 15% were <20 years old, 31% were married, 28% had less than an elementary school certificate, and 35% were primiparous (Table 1). Among those who had attended at least one visit prior to their current visit, 96% reported having been tested for HIV and 85% having been tested for syphilis. Among those tested, results had been returned to >90% for both. Overall most women reported having received most standard clinical services (see Table 2 for list of standard clinical services) during their visit (98% had blood pressure taken, 97% were weighted, 80% had fetal heart tones checked). In contrast, relatively few women reported receiving standard counseling messages (see Table 2 for list of standard counseling services; 42% given estimated due date, 27% counseled on delivery needs, 44% counseled on danger signs). Overall 26% of women waited ≥4 h to be seen, and 28% spent ≤5 min with their provider. Most women reported having felt respected during their visit (98%) and as if their provider cared about them (93%).

**Table 1:** Demographic characteristics of study participants

	Antenatal		Postnatal	
	*n* = 867		*n* = 64	
	*n*	%^a^	*n*	%^a^
Maternal age (y)				
≤ 20	124	14.5	11	17.1
21–25	205	23.9	17	26.5
26–30	286	32.5	16	25.1
≥ 31	252	29.0	20	31.4
Marital Status				
Married	277	31.3	19	29.8
Not married, cohabiting	451	52.3	31	48.4
Not married, not cohabiting	139	16.4	14	21.8
Education				
No elementary school certificate	241	28.2	16	24.8
Elementary school certificate	89	10.0	5	7.8
Some secondary, no certificate	394	45.6	31	48.6
Finished secondary or more	143	16.0	12	18.8
Previous births				
No previous births	302	34.8	31	48.3
1 previous birth	252	29.0	15	23.4
2–3 previous births	235	27.1	9	14.0
≥ 4 previous births	78	9.1	9	14.3
Location of most recent delivery				
Health facility	–		44	74.6
Home	–		16	25.4
Unknown	–		4	6.3
Experienced delivery complications	–		15	23.4

^a^Frequencies expressed as weighted percentages

**Table 2:** Frequency of visit services reported among women seeking antenatal or postnatal health services in Haiti

	Antenatal		Postnatal	
	*n* = 867		*n* = 64	
	*n*	%^a^	*n*	%^a^
Services received during pregnancy^b^				
Tested for HIV	560	95.9	–	
Received HIV results^c^	520	93.0	–	
Tested for Syphilis	496	85.0	–	
Received Syphilis results^d^	462	93.3	–	
Current Visit				
Visit number				
1	264	29.7	42	66.5
2	208	24.0	14	22.0
3	157	18.1	3	4.7
4	100	11.9	–	
5+	117	14.0	–	
Unknown	21	2.3	5	7.8
Clinical services received				
Blood pressure taken	846	97.6	60	93.8
Abdomen examined	682	79.3	50	78.2
Weighed	843	97.1	–	
Babies heartbeat checked^e^	402	80.1	–	
Scheduled next appointment	705	81.6	–	
Appointment written down^f^	650	92.6	–	
Asked about well being	–		60	93.8
Counseling services received				
Nutrition counseling	524	61.8	–	
Given estimated due date	348	41.9	–	
Discussed how baby is growing	340	40.2	–	
Counseled on delivery needs	225	27.4	–	
Advised to deliver in a facility	687	80.0	–	
Discussed birth plan	195	23.3	–	
Counseled on delivery danger signs	372	44.1	–	
Counseled on pregnancy danger signs	416	49.0	–	
Counseled on postpartum family planning	288	33.4	–	
Counseled on birth interval	148	18.5	–	
Discussed return to sexual activity	–		33	51.5
Discussed breastfeeding	–		48	75.0
Discussed pregnancy prevention	–		35	54.6
Had maternity card at visit	292	36.1	22	34.3
Satisfaction				
Wait time				
< 1 h	141	16.3	21	32.9
1- < 2 h	172	20.1	12	18.6
2- < 3 h	171	19.9	7	11.0
3- < 4 h	151	17.4	13	20.3
≥ 4 h	227	25.7	11	17.2
Missing/Unknown	5	0.6	0	0.0
Total time with providers				
≤ 5 min	254	28.2	18	28.2
6–10 min	242	28.2	16	24.9
11–15 min	171	20.3	14	21.8
≥ 16 min	186	21.8	15	23.5
Missing/Unknown	14	1.6	1	1.6
Satisfied with providers				
Felt respected	849	98.0	63	98.4
Felt provider cared	799	92.6	61	95.3

*Abbreviations*: *HIV* human immunodeficiency virus, *hrs* hours, *min* minutes, *DOB* date of birth

^a^Percentages are expressed as weighted frequencies

^b^Among those on visit 2 or more (*n* = 582)

^c^Among those tested for HIV (*n* = 560)

^d^Among those tested for Syphilis (*n* = 496)

^e^Among those of gestational age ≥5 months (*n* = 507)

^f^Among those with a scheduled appointment (*n* = 705)

### Postnatal care

Out of 77 postpartum women presenting for postnatal care during study days, 12 declined participation yielding a response rate of 84.4%. Out of the 65 women interviewed, 1 woman was excluded for missing information on marital status resulting in a final analytic sample of 64 postpartum women. Among the remaining 64 postpartum women, 17% were <20 years old, 30% were married, and 25% had less than an elementary school certificate. Approximately half were seeking postpartum care after the delivery of their first child and 23% reported experiencing a delivery complication. In addition, 75% reported having delivered their most recent child in a health facility (Table 1). Among postpartum women seeking postnatal care, 67% were attending their first visit (median: 7 days since delivery (Interquartile Range (IQR): 0–11 days); Table 2). Overall, most women reported having received standard clinical services during their visit (94% had blood pressure taken, 78% had abdomen examined, 94% were asked about wellbeing). In contrast, relatively fewer women reported receiving standard counseling services (52% discussed return to sexual activity, 55% discussed pregnancy prevention). Overall 17% of women waited ≥4 h to be seen, and 28% spent ≤5 min with their provider. Most women reported having felt respected during their visit (98%) and as if their provider cared about them (95%).

### Maternal knowledge

Among pregnant women seeking antenatal care, only 23% knew the recommended birth interval and 39% of women were unable to name a single modern method of contraception (Table 3). Among women seeking postnatal care, 67% knew the recommended duration of exclusive breastfeeding but 81% were unable to name any component of the lactational amenorrhea method of contraception.

**Table 3:** Knowledge on family planning, birth spacing and nutrition among women seeking antenatal or postnatal services

	Number	Percent^a^
Among women seeking antenatal care (*n* = 867)		
Knew recommended birth interval^b^	197	23.2
Post-partum family planning methods^c^		
Lactation amenorrhea	59	6.9
Intrauterine device	6	0.7
Depo Provera (Injectables)	399	46.7
Male condom	147	17.9
Progestin Implant	86	10.8
Birth Control pills	247	29.8
Knew none	343	38.7
Among women seeking postnatal care (*n* = 64)		
Knew recommended duration for exclusive breastfeeding^d^	43	67.2
Spontaneously reported the following components of LAM		
Breastfeeding on demand	8	12.5
No menstrual period	1	1.6
Exclusively breastfeed	2	3.1
Baby <6 months	6	9.4
Knew all four components of LAM	0	0.0
Didn’t know any component of LAM	52	81.3

*Abbreviations*: *mos* months, *LAM* locational amenorrhea method

^a^Percentages expressed as weighted frequencies

^b^A spontaneous answer of 2 or 3 years was considered correct

^c^Spontaneous answers were recorded, and more than 1 answer was possible from each pregnant woman

^d^A spontaneous answer of 6 months was considered correct

### Counseling received during antenatal care visits

**Fig. 1:**
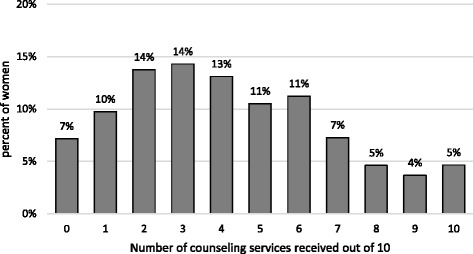
Weighted percentage of number of counseling services reported having been received by pregnant women seeking antenatal care out of 10 (*n* = 867)

Figure 1 displays the overall percent of pregnant women who received the corresponding number of counseling message during their antenatal visit out of the 10 counseling messages evaluated. Only 5% of women received all 10, and 7% did not recall receiving any. Overall, 42% of women reported receiving at least 5 out of 10 counseling messages. Pregnant women who were on a repeat visit had increasingly greater odds of having received five out of 10 counseling messages (2^nd^ visit: adjusted odds ratio [aOR] = 1.71, 95% confidence interval [CI] 1.09–2.67; 5+ visit: aOR = 5.36, 95% CI 2.87–10.01; Table 4), compared with pregnant women who were on their first visit. Additionally, women who had an elementary school certificate or had finished secondary school also had higher odds of having received five out of 10 counseling messages (elementary school certificate: aOR = 1.96, 95% CI 1.12–3.42; finished secondary school or more aOR = 1.96, 95% CI = 1.09–3.07), compared with women who had no elementary school certificate.

**Table 4:** Factors associated with receiving at least 5 out of 10 counseling messages among women seeking antenatal services (*n* = 867)

	*n*	% Who received at least 5/10 messages^a^	OR	95% CI	aOR^b, c^	95% CI
Prenatal visit number^d^						
1	264	21.7	1.00	–	1.00	–
2	208	36.9	2.10	1.39–3.19	1.71	1.09–2.67
3	157	48.2	3.35	2.17–5.18	2.55	1.51–4.31
4	100	54.0	4.22	2.57–6.94	2.63	1.43–4.83
5+	117	70.3	8.54	5.19–14.04	5.36	2.87–10.01
Maternal age (y)						
≤ 20	124	36.3	1.00	–	1.00	–
21–25	205	37.2	1.04	0.65–1.66	0.94	0.54–1.64
26–30	286	41.3	1.24	0.80–1.92	1.10	0.61–2.00
31	252	49.4	1.72	1.10–2.68	1.62	0.87–3.02
Marital status						
Married	277	42.5	1.00	–	1.00	–
Not married, cohabiting	451	40.2	0.91	0.67–1.23	1.00	0.69–1.45
Not married, not cohabiting	139	46.6	1.18	0.78–1.79	1.46	0.88–2.42
Education						
No elementary school certificate	241	37.7	1.00	–	1.00	–
Elementary school certificate	89	46.6	1.44	0.88–2.37	1.96	1.12–3.42
Some secondary, no certificate	394	41.3	1.16	0.83–1.62	1.32	0.88–1.97
Finished secondary or more	143	48.2	1.53	1.00–2.34	1.83	1.09–3.07
Gestational age						
< 5 months	360	28.8	1.00	–	1.00	–
≥ 5 months	507	51.1	2.59	1.93–3.46	1.36	0.91–2.02
Parity						
Primiparous	302	38.5	1.00	–	1.00	–
Multiparous	565	43.8	1.24	0.93–1.66	1.12	0.76–1.67

*Abbreviations*: *y* years, *mos* months, *CI* confidence interval

^a^Overall percentages are expressed as weighted percentages, but counts are unweighted

^b^Odds ratios and 95% confidence intervals were calculated using weighted logistic regression

^c^Adjusted odds ratio (aOR) adjusted for department, prenatal visit number, maternal age, marital status, maternal education, gestational age, and parity

^d^Due to the low number of women with unknown visit number (*n* = 21), the corresponding odds ratios and confidence intervals are suppressed

### Visit frequency for ANC

The number of charts reviewed varied by facility (*n* = 85–392) for a total of 1,411 antenatal charts. Among the reviewed charts, 27% indicated only a single visit, 19% indicated 2 visits were completed, 17% indicated 3 visits were completed, 17% indicated 4 visits were completed, and 21% indicated 5 or more visits were completed (data not shown).

### Provider knowledge

A total of 39 interviews were conducted with health care providers, and all providers who were approached consented to be interviewed, however, one provider was excluded due to missing information on which services he provided. Of the 38 healthcare providers who completed a knowledge assessment questionnaire and reported which services they provided, 3 were doctors, 5 were midwives, 18 were nurses, 10 were auxiliary nurses and 2 were students (Table 5). Twenty-nine (76%) of the 38 providers provided both prenatal and postnatal care, while 8 (21%) reported only providing prenatal care and one (3%) reported providing only postnatal care; one (3%) observation was missing. Nearly two-thirds (66%) of the providers had been practicing for 3 or more years. Overall, 49% knew the recommended number of antenatal care visits a pregnant women should have, 65% were able to name three methods of postpartum family planning and 70% were able to name 3 delivery danger signs. Overall, 67% knew the recommended number of postnatal care visits, 57% were able to name 3 postpartum danger signs, and 53% knew 3 contraception methods that could be offered immediately after delivery.

**Table 5:** Provider knowledge on antenatal and postnatal care components (*n* = 38)

	Number	Percent^a^
Provider Type		
Doctor	3	7.9
Midwife	5	13.2
Nurse	18	47.4
Auxiliary Nurse	10	26.3
Student	2	5.3
Services Provided		
ANC Only	8	21.1
PNC Only	1	7.9
ANC and PNC	29	76.3
Antenatal Care Visit Info^b^		
Knew recommended total # ANC visits	18	48.7
Knew recommended timing for all visits	9	24.3
Antenatal Care Counseling Knowledge^b^		
Knew when to start discussing postpartum family planning	23	62.2
Named three postpartum family planning methods	24	64.9
Knew all four components of LAM	5	13.5
Said exclusively breastfeeding	29	78.4
Said baby < 6 months	24	64.9
Said no period	22	59.5
Said breastfeeding on demand	14	37.8
Knew postpartum birth interval^c^	31	83.8
Knew 3 delivery danger signs	26	70.3
Knew delivery location risk	30	81.1
Postnatal Care Visit Info^d^		
Knew recommended total # PNC visits	20	66.7
Knew recommended timing for all Visits	0	0.0
Postnatal Care Counseling Knowledge^d^		
Knew to examine both baby and mom	22	73.3
Knew 3 postpartum danger signs	17	56.7
Knew which body temperatures were abnormal	7	23.3
Knew 3 contraception methods that could be offered immediately after delivery	19	63.3
Birth Control Pills	16	53.3
Depo Provera (Injectables)	11	36.7
Male Condom	14	46.7
Lactation amenorrhea	18	60.0
Intrauterine Device	3	10.0
Implant	4	13.3
Tubal Ligation	4	13.3
Natural Family Planning	3	10.0
Didn’t know any types	1	3.3

*Abbreviation*s: *ANC* antenatal care, *LAM* lactational amenorrhea method, *PNC* postnatal care

^a^Percentages expressed as unweighted frequencies

^b^Only includes those providers who provide antenatal care (*n* = 37)

^c^An answer of 2 or 3 years was considered correct

^d^Only Includes those providers who provide postnatal care (*n* = 30)

## Discussion

Results from a sample of pregnant and postpartum women seeking care at large health facilities in three departments in Haiti suggest underutilization of both antenatal and postpartum services. Additionally, while routine antenatal clinical services, such as blood pressure checks, were systematically provided, education messages were reported to occur far less frequently and knowledge of key life-saving measures, such as danger signs of pregnancy and postpartum contraceptive methods was low among pregnant and postpartum women. Uptake of antenatal care has been linked to a reduction in maternal morbidity and mortality and improvement in neonatal survival [6, 7]. Antenatal care represents a point of contact between providers and pregnant women at which key antenatal services and educational messages may be given [8]. Antenatal care components can identify potential complications in pregnancy and counseling can help women recognize danger signs of pregnancy, understand the benefits of facility deliveries, and the importance of healthy spacing of pregnancies through the use of postpartum contraception [9]. Likewise, postpartum care is an opportunity to identify complications after delivery, and to provide health promotion messages and preventive services, such as family planning methods to prevent a rapid repeat pregnancy and improve both maternal and newborn survival [10]. The low level of delivery and or retention of educational messages among our sample participants is concerning and emphasizes the importance of standardizing and delivering quality antenatal care and measuring quality indicators alongside quantity of antenatal care visits [see ref in comment] [11].

Review of 1,141 antenatal charts of recently delivered women demonstrated that 27% of our sample attended only a single antenatal care visit and 47% attended two visits or less. Furthermore, only 36% of women attended the recommended 4 antenatal care visits. This is far less than the national estimate of 67% of women who report attending at least 4 antenatal visits [3]. While it is possible that some women may have attended a portion of their antenatal care visits at another facility, therefore not recorded in the charts reviewed, this number is likely to be low. Alternatively, regional differences in antenatal care uptake or desire to report higher uptake in the national Demographic Health Survey may explain some differences. It is both a global and national recommendation that pregnant women deliver in a health facility with skilled care, yet the overall facility delivery rate in Haiti is low at 36% [3]. Attendance at ANC is a determinant of a facility delivery [4], and under-utilization of antenatal care, even among women who do have a facility delivery, has been shown to increase the risk of poor pregnancy and neonatal outcomes [6]. Further efforts to increase uptake of antenatal care and examine barriers to seeking care among should be considered.

The majority of pregnant women seeking antenatal care received the recommended clinical services such as HIV testing, blood pressure, weight checks and fetal viability assessments. In contrast relatively few women received adequate counseling messages. For example, 44% reported receiving education on danger signs of pregnancy and 18% on the importance of birth spacing for maternal and newborn health. Antenatal care is an opportunity to provide services, screen for complications, and counseling at multiple time points during pregnancy [2, 4]. Components of counseling should include education on danger signs of pregnancy, birth preparedness, breastfeeding, and the importance of postpartum family planning for birth spacing A total of 10 counseling messages was expected at each visit. Among our sample of women seeking antenatal care, only 5% reported receiving all 10 messages and 7% reported they received none of the messages, indicating that a critical opportunity to provide key health messages to pregnant women was missed. An alternative explanation to the low report of counseling messages may be a lack of understanding or generalized low health literacy among the study population. This hypothesis is supported by our findings which show women of higher educational attainment and women who were at a repeat visit (compared to women attending their first antenatal care visit) were more likely to report receipt of 5 or more out of 10 counseling messages. It is possible that higher education and/or repeat exposure to the counseling messages may aids in understanding and suggesting that implementation of simple scripted counseling messages that could be repeated at every antenatal visit may improve comprehension and retention. Interestingly 28% spent ≤ 5 min with their provider which may be another contributing factor to the low report of counseling received. While time with the providers was low, clinic wait times were very high (26% waited >4 h) therefore providing scripted messages with a lay counselor in the waiting area may also improve retention and comprehension.

A woman’s ability to achieve recommended birth spacing can improve both maternal and neonatal outcomes [10]. This is best achieved by adopting a family planning method in the immediate postpartum period. Use of family planning postpartum among many Haitian women is low where only 23% of women reporting use of any family planning method in the first months, yet only 2% of postpartum Haitian women desire subsequent conception in the next 2 years after birth [12] and desire among recently delivered women living in Northern Haiti to adopt a family planning method postpartum is high [13]. Our study demonstrated low attendance at postpartum care visits highlighting the need to discuss postpartum family planning during the antenatal care period. Limited knowledge of postpartum family planning methods by both women and providers were also observed. Provider knowledge and comfort with family planning methods can influence uptake of family planning methods by women [14]. A concerted effort towards improved provider knowledge and comfort with family planning methods postpartum could be explored as a strategy to meet the known demand for delaying next birth among Haitian postpartum women.

This study is the first to characterize antenatal and postpartum care in Haiti through a quality of care lens. Our methodology allowed us to triangulate findings from several sources, including providers, patients and hospital records, in order to strengthen the validity of our findings, however, our study has several limitations. First we limited our study to large health facilities, limiting the generalizability of our findings. Second, we sampled women seeking antenatal and postnatal care at facilities located in 3 departments within Haiti and therefore findings may not be nationally generalizable as these departments were not chosen at random. Third, we surveyed patients immediately after receiving care, which minimizes recall bias, however may have increased the likelihood of desirability bias among patients. Fourth, antenatal care visit numbers abstracted from charts may be lower than recommended as a result of preterm delivery or through seeking antenatal care at multiple facilities, neither explanation this study was designed to address. Finally, although our provider sample was small, it was an exhaustive sample and therefore represents the current knowledge held by antenatal care providers at large hospitals in the three departments, but is not generalizable to the larger national population of providers.

## Conclusions

Our study suggests that antenatal and postnatal care uptake in Haiti remains low. Despite frequent report of the provision of standard antenatal and postnatal clinical services (such as blood pressure checks, weight recording, and provision of HIV testing), report of having received standard recommended counseling messages remains low. Efforts to improve quality of antenatal and postnatal care should include a focus on increasing the provision of standardized counseling messages at all antenatal care visits, reiterating the importance of seeking postpartum care and utilizing postpartum family planning to ensure health birth spacing, and ensuring provider knowledge of antenatal and postpartum care is current with national and WHO recommendations. The consistent provision of standardized counseling recommendations coupled with regular health provider trainings is recommended to improve quality of antenatal and postnatal care in Haiti and further reduce maternal mortality.

## Abbreviations

ANC: Antenatal care
aOR: Adjusted odds ratio
CDC: Centers for Disease Control and Prevention
CI: Confidence interval
IQR: Interquartile range
MESI: Monitoring, evaluation and surveillance intégrée
MMR: Maternal mortality ratio
ODK: Open data kit
PNC: Postnatal care
WHO: World Health Organization
