# Erratum to: Quality and uptake of antenatal and postnatal care in Haiti

**DOI:** 10.1186/s12884-017-1258-z

**Published:** 2017-03-14

**Authors:** Kelsey R. Mirkovic, Eva Lathrop, Erin N. Hulland, Reginald Jean-Louis, Daniel Lauture, Ghislaine Desinor D’Alexis, Endang Handzel, Reynold Grand-Pierre

**Affiliations:** 1 Epidemic Intelligence Service, Centers for Disease Control and Prevention, Atlanta, GA USA; 2 Division of Global Health Protection, Centers for Disease Control and Prevention, Atlanta, GA USA; 3 Division of Global Health Protection, Centers for Disease Control and Prevention, Port-au-Prince, Haiti; 4 Haiti Ministry of Public Health and Population, Port-au-Prince, Haiti

## Erratum

In the original publication of this article [1], the author Endang Hanzel was written incorrectly. The correct spelling should have been Endang Handzel. The original article has now been updated to reflect this change.
